# Assessing Post-Driving Discomfort and Its Influence on Gait Patterns

**DOI:** 10.3390/s21248492

**Published:** 2021-12-20

**Authors:** Marko M. Cvetkovic, Denise Soares, João Santos Baptista

**Affiliations:** 1 Associated Laboratory for Energy Transports and Aeronautics (PROA-LAETA), Faculty of Engineering, University of Porto, 4200-465 Porto, Portugal; m.cvetkovic-3@tudelft.nl (M.M.C.); Denise.Soares@aum.edu.kw (D.S.); 2 Cognitive Robotics Department, Faculty of Mechanical, Maritime and Materials Engineering, Delft University of Technology, 2600 AA Delft, The Netherlands; 3 Liberal Arts Department, American University of the Middle East, Egaila 15453, Kuwait

**Keywords:** drivers monitoring, prolonged sitting, interface pressure, driving posture, musculoskeletal disorders, gait parameters

## Abstract

Professional drivers need constant attention during long driving periods and sometimes perform tasks outside the truck. Driving discomfort may justify inattention, but it does not explain post-driving accidents outside the vehicle. This study aims to study the discomfort developed during driving by analysing modified preferred postures, pressure applied at the interface with the seat, and changes in pre- and post-driving gait patterns. Each of the forty-four volunteers drove for two hours in a driving simulator. Based on the walking speed changes between the two gait cycles, three homogeneous study groups were identified. Two groups performed faster speeds, while one reduced it in the post-steering gait. While driving, the pressure at the interface and the area covered over the seat increased throughout the sample. Preferred driving postures differed between groups. No statistical differences were found between the groups in the angles between the segments (flexed and extended). Long-time driving develops local or whole-body discomfort, increasing interface pressure over time. While driving, drivers try to compensate by modifying their posture. After long steering periods, a change in gait patterns can be observed. These behaviours may result from the difficulties imposed on blood circulation by increasing pressure at this interface.

## 1. Introduction

The automotive industry has continually enhanced vehicle interior and exterior designs to increase the transport’s comfort, efficiency, and reliability. Nonetheless, injuries [1], work-related musculoskeletal disorders [2], and even perceived discomfort [3] are still present among professional drivers. De Looze et al. [3], and later Hiemstra-van Mastrigt et al. [4], created models and explained the interaction between drivers’ anthropometric characteristics, physical vehicle features and work-related tasks to describe driving comfort/discomfort. However, even adapting drivers’ working environment to a wider range of individuals, prolonged awkward driving strategies must be avoided. Considering exposure time when assessing observed discomfort is crucial, knowing that discomfort or pain while driving can arise and increase with time [5].

Driver’s seat must be adjustable and create a feeling of well-being, accommodating and supporting around 70% of the total body weight [6]. The interface pressure at the seat pan is mainly generated by bony prominences (ischial tuberosities, sacral coccygeal area, and greater trochanter) and the lower limbs, muscles, and tendons [7]. For instance, the interface pressure under ischial tuberosities bones could be up to 11 times greater [7] compared to the rest of the buttocks/thigh surface, with the covered area by 18% [8]. Uneven pressure distribution in the lower limbs area with a higher dynamic load on the musculoskeletal system will manifest as a perception of pain, fatigue, soreness, tensions, and numbness [9].

Individuals with different anthropometric attributes may have experienced different discomfort ratings [10]. The percentage of fat tissue and the distance between ischial tuberosities bones appears to be an essential factor considering interface pressure between subject and seat [10]. There may be a difference between genders since females tend to have a higher percentage of fat in the lower limbs area and more significant distance between the bony prominence in the pelvic area [11]. In contrast, a person having a sharper ischial tuberosity is more at risk of developing disorders caused by prolonged sitting [12].

Apart from work-related musculoskeletal disorders developed over the driving time, acute work-related injuries caused by slips, trips, and falls are often correlated with professional drivers [1]. Xia et al. [13] investigated the occurrence of injuries and diseases caused by non-driving tasks among occupational drivers. As a mechanism of injuries, slips, trips, and falls were reported in the range from 15 to 25% (with the highest prevalence was among automobile drivers). It should be noted that all injuries were occurred walking on the same or different ground levels [13]. Gait performance and postural stability depend on a complex relationship between the musculoskeletal, neurological, vestibular, somatosensory, and visual systems [14]. Disturbing one of the mentioned systems, human gait performances might change gait patterns, causing falls [15] or slips [16].

Moreover, subjects with developed fear or risk of falling might reduce walking speed (WS), adopting shorter step length (SL), decreasing the range of motions in lower limb joint angles, prolonging stance phase (SF) and shortening swing phase (SWF) [17]. The fact is that the risk of falls is increased with age, whereas older subjects are more prone to fall [18]. Regarding this statement, older drivers might be at higher risk of acute injuries outside vehicles [19]. 

The primary purpose of this study is to investigate if prolonged driving can influence the gait pattern. The specific goals of this research are the following: (a) to reveal whether the subjects can be classified by the pattern of changing in WS and if changes of WS influence defined gait variables; (b) to examine whether the differences between groups in walking strategies are related to different postures (angles between body segments) and whether, as a result, they apply different interface pressures over the seat pan; (c) to define whether the driving discomfort was developed, based on exceeded interface pressure values and repositioned driving angles.

## 2. Materials and Methods

### 2.1. Participants

Forty-four subjects (22 female and 22 males) aged between 20 and 40 years old volunteered and agreed to participate in the study. None of the participants had any surgical intervention, neurological disorder, or musculoskeletal injury in the lower limbs in the past twelve months. Unusual walking patterns, inability to perform barefoot or shod walk without assistance, poor balance, and incompetence to steer a driving simulator in a demanded virtual environment were considered as exclusion criteria. Before the experimental process, informed consent was provided to every participant. This study was submitted and approved by the Ethics Committee of the University of Porto and registered under No84/CEUP/2019.

### 2.2. Instruments

Spatiotemporal variables were recorded using Walkway Pressure Assessment Systems (Tekscan^®^, Boston, MA, USA). The sampling rate was defined at 100 Hz, with the pressure range from 1 to 850 kPa. The mat calibration was accomplished before the data acquisition according to the manufacturer’s instructions and the Tekscan^®^ software—Walkway version 7.02.

Six Logitech C920 web-cameras (Logitech, Newark, NJ, USA) were used to record preferred driving postures in full HD resolution (1920 × 1080 pixels), with a sampling rate at 30 Hz. Cameras were positioned and secured on tripods with 60 to 120 degrees between each, taking the centre of the drivers’ seat as the reference point. Before each experiment, the camera calibration was done using a rigid cube placed on top of the drivers’ seat pan.

### 2.3. Driving Simulator

The driving simulator was a modified Volvo 440 turbo vehicle installed with sensors in the steering wheel, pedals, ignition key, and other technical elements. The virtual environment was set to mimic the car movements on a two-lane highway. It included other vehicles’ actions and behaviours in both traffic directions, with 60 frames per second refresh rate.

### 2.4. Equipment Synchronisation

Each volunteer was instructed to press a specific region/button of the Tactilus^®^ mat to define a common event from which the data acquisition was synchronised. This action triggered an LED light seen by all recording cameras while providing an input to the sensing mat. 

### 2.5. Characterisation of Driving Discomfort

Interface pressure distribution is often used as an objective measure to determine variation between comfort and discomfort among different seats and materials and a proven method for quantifying perceived comfort/discomfort [3,4,20]. Also, based on previous studies [21,22], the interface pressure threshold was defined at 4.3 kPa applied over the total seat area. Above this value, a perceived local or total bodily discomfort would occur. Furthermore, suppose the pressure at the interface exceeds 6.4 kPa under the buttocks area or exceeds 3 kPa under the thighs area. In that case, it will cause tissue ischemia and affect sitting comfort [23].

Frequently repositioning preferred driving postures and perceived body discomfort have proven their correlation [9] and will be considered a method to conclude developed distress over the driving time.

### 2.6. Procedures

Participants’ age, body height, weight, and driving experience were recorded before data collection. Eight reflective markers, with 2 cm diameter, were positioned on anatomical landmarks corresponding to major joints: lateral metatarsal head (foot), lateral and medial malleolus (ankle), lateral and medial epicondyle of the femur (knee), great trochanter (hip), right and left acromion (shoulder), lateral epicondyle of the humerus (elbow), and radial styloid process and heel of the ulna (wrist). The position of the markers allowed to record the preferred driving postures by calculating the main joint angles.

Spatial and temporal data were acquired in two different periods—before and after steering the driving simulator. The gait was performed on a marked pathway where the pressure mat was included. The distance between the start line and the pressure mat enabled sufficient space for a participant to perform two steps before contacting the mat mentioned above. Participants were instructed to walk at a self-selected and comfortable pace before and after finishing the driving tasks. Three valid and complete gait cycles were performed for each assessment period.

The driving session demanded constant steering of the driving simulator for 120 min (about half a work shift), with an average driving speed of 90 km/h (maximum road speed limit in most countries). 

Entering the driving simulator, participants adjusted the interior distances of the seat according to their individual preferences. The interior dimensions could be adjusted on horizontal points (changing distance between the heel and hip point) and backrest angles, respecting the SAE J1100 [24]. The horizontal and vertical distances of the steering wheel position could not be modified, being the same for all participants. The first five minutes of steering were intended for familiarisation with the virtual environment. During the driving session, variables based on flexion/extension of main joint angles (ankle, knee, hip, shoulder, elbow, and wrists), applied interface pressure, and the covered area on the seat pan was acquired. A 10-s recording was performed at the 5th and 120th minute of the driving session.

### 2.7. Data Processing

Both temporal and spatial gait parameters were collected and analysed using Walkway software, version 7.02 (Tekscan^®^, Boston, MA, USA). An average value of the three gait trials (pass over the Walkway Pressure Assessment System) per walking period was further exported to Excel 2016 (Microsoft Corporation, Washington, DC, USA) spreadsheet. 

Driving posture data were processed with the software SkillSpector^®^ version 1.3.2. (Video4Coach, Odense, Denmark). The biomechanical model was formed by linking reflective markers placed on the subject’s body. The direct linear transformation was precisely linked with the previously defined calibration. It was used to calculate the coordinates and, therefore, to create the model. The left side of the driver’s posture, with defined driving angles [25], was analysed considering the flexion/extension of the main joint angles.

**Figure 1:**
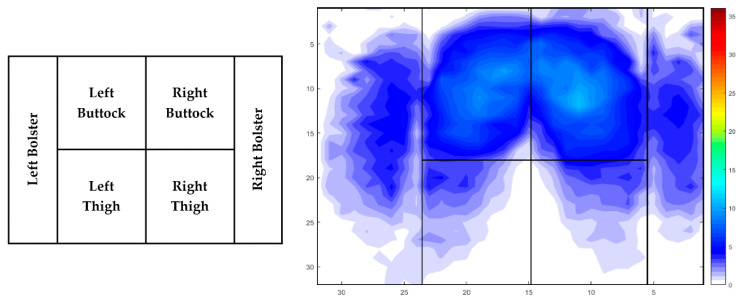
Definition of lower limbs area.

Applied interface pressure and covered area on the drivers’ seat pan were recorded using Tactilus^®^ software, version 8.1 (Tactilus^®^, New York, NY, USA). Two-dimensional data referred to the entire seat pan surface was further assessed using the same software. Average interface pressure represents an average peak pressure recorded during the whole surface area. The contact area was defined by summarising activated cells of the interface pressure equipment triggered during the driving session. Further examination of applied average interface pressure was assessed for the following lower limb area: left bolster (LB), left buttock (LBU), left thigh (LTH), right buttock (RBU), right thigh (RTH), right bolster (RB) (Figure 1). Selected areas were modified for each participant separately, considering different anthropometric attributes.

### 2.8. Statistical Analysis

An absolute value, calculated from WS differences (difference = post-steering WS—pre-steering WS), was further implemented as a single variable into the Hierarchical clustering method to divide the study population into WS subgroups. From this point, one of the hierarchical clustering procedures, Ward’s method, was chosen to define approximately equal subgroups members based on WS alternation between two gait periods. The subgroups were classified based on visual assessment of the obtained clusters presented in the dendrogram respecting the Euclidean distance. The Shapiro-Wilk test was performed to investigate whether the data was (not)normally distributed. Furthermore, One-Way ANOVA was applied to examine whether the between-group comparison differs concerning personal characteristics. The Repeated Measures ANOVA with Tukey’s honest significance test was performed to investigate the main effect of time on spatiotemporal (before vs. after), interface pressure variables (5th vs. 120th minute of driving), and preferred driving postures (5th vs. 120th minute of driving). If a significant time effect was found, the pairwise comparison with Bonferroni adjustment was accomplished. The critical significant threshold was defined at α ≤ 0.05.

## 3. Results

### 3.1. Description of the Sample Size Based on the Cluster Classification

The Wards’ method identified three clusters based on an absolute value obtained on WS alteration. Under cluster 1 (C1) and cluster 2 (C2) were selected participants with the increased post-steering WS. Subjects categorised under cluster 3 (C3) reduced WS after 120 min of the driving process. Differences in pre-and post-driving WS were recognised for all subgroups (*p* < 0.001) using Repeated Measures ANOVA.

The remaining variables, such as anthropometric attributes, driving experience, and the time spent steering a vehicle per week was not established as significantly different among the subgroups (Table 1).

### 3.2. Influence of Prolonged Driving on Spatiotemporal Characteristics

Spatial and temporal data differ between the subgroups in gait performed in two walking periods (Table 2). The C1 adopted slightly (based on mean differences) faster-WS (*p* < 0.001) with a higher Cadence (CA) (*p* = 0.002). Therefore, the walking strategy resulted in a shorter TODST (*p* = 0.002). Statistical analysis indicated similar changes with the C2 subgroup, but with one difference. The C2 performed post-steering gait with higher WS (*p* < 0.001) and higher CA (*p* < 0.001), and consequently, this walking strategy resulted in a faster Gait cycle time (GCT) (*p* < 0.001) with lesser Terminal double support time (TDST) (*p* < 0.001) and Total Double support time (TODST) (*p* < 0.001). Decreased WS (*p* < 0.001) among the C3 participants culminated in lesser CA (*p* = 0.041) and prolonged TDST (*p* = 0.002). Moreover, the newly selected walking strategy prolonged TODST (*p* < 0.001) and increased the GCT as well (*p* = 0.010). Summarised gait outcomes accomplished before the driving process (Table 2) did not indicate a statistically considerable difference between the subgroups. The change in the walking strategy after steering showed a significant effect on the results between C2 and C3, resulting in slower WS (*p* = 0.004) with prolonged GCT (*p* = 0.016), TDST (*p* = 0.026), and TODST (*p* = 0.013) among the C3 participants.

Additionally, the multiple comparisons with applied Bonferroni correction also uncovered that C2 participants increased SF (*p* = 0.044) and SWF (*p* = 0.044) compared to the C3 subgroup. A comparison between participants with increased post-steering gait showed that the C2 had a higher WS (*p* = 0.004) and CA (*p* = 0.038) than the C1 subgroup, but there were no differences in the remaining spatiotemporal variables.

### 3.3. Postures during Prolonged Driving

**Figure 2:**
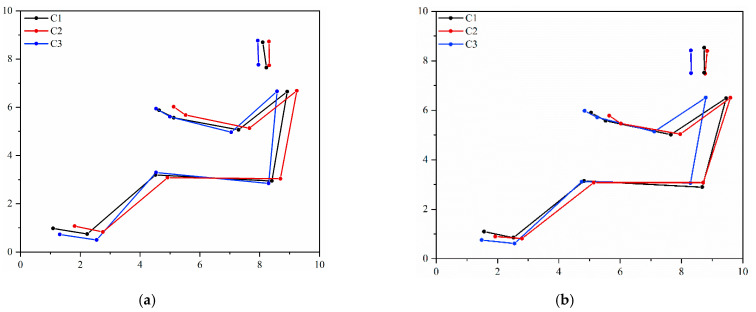
Illustration of the preferred driving postures between the subgroups at the initial and final recording. Note. C1—Cluster 1; C2—Cluster 2; C3—Cluster 3. (**a**) 5th minute of steering; (**b**) 120th minute of steering.

Transition in self-selected driving strategies between two driving periods is displayed in Figure 2. Adopted driving postures by C2 and C3 participants did not change between the two recording periods (5th and 120th minute of constant driving), while preferred driving angles among the subgroup C1 were affected only in the wrist (*p* = 0.044), extending it during the drive.

Preferred driving angles among the subgroups do differ (Figure 2). C3 subjects adopted flexed hip (*p* = 0.005) and knee (*p* = 0.049) postures at the initial recording compared to the C2 subgroup. There was no significant between-group difference in preferred driving postures among subgroups after 120 min of driving.

### 3.4. Average Interface Pressure and Covered Area on the Drivers’ Seat Pan

#### 3.4.1. Interface Pressure Variables by Total Seat Pan Area

**Figure 3:**
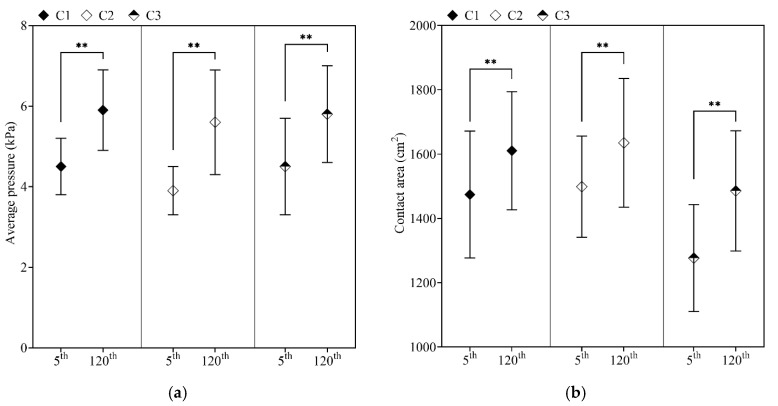
Interface pressure variables of the total seat pan area during prolonged driving. Note. C1—Cluster 1; C2—Cluster 2; C3—Cluster 3; 5th minute of driving; 120th minute of driving; Statistically significant difference is indicated as ** (*p* < 0.001). (**a**) Average interface pressure in the minutes 5th and 120th; (**b**) Average contact area in the minutes 5th and 120th.

The mean of interface pressure variables and a significant difference between the two periods are illustrated in Figure 3. The time spent steering the driving simulator affected contact area (all *p* < 0.001) and applied interface pressure (all *p* < 0.001), linearly increasing it over the driving time. Noteworthy, participants with decreased WS (C3) covered lesser seat pan surface area at the initial recording compared with faster (C2; *p* = 0.045) and slightly faster (C1; *p* = 0.034) walking subgroups.

#### 3.4.2. Average Interface Pressure by Defined Zones

**Figure 4:**
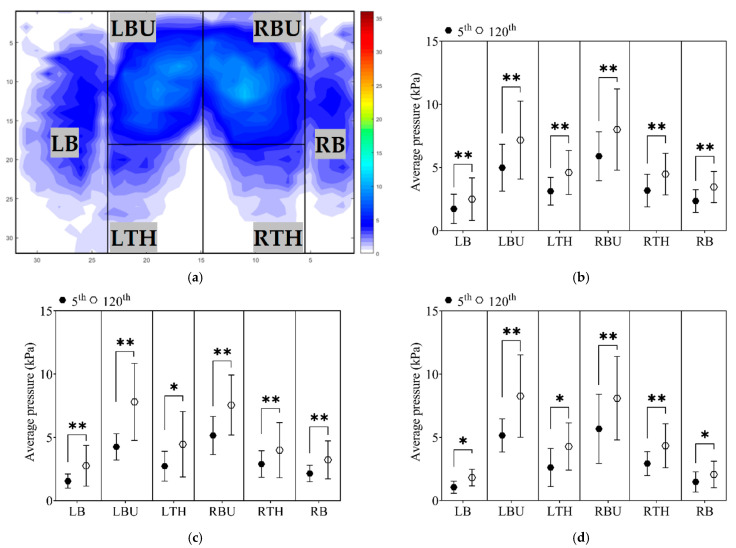
Applied average interface pressure by cluster groups with indicated statistical difference. (**a**) Definition of the lower limbs zones; (**b**) Cluster 1 group; (**c**) Cluster 2 group; (**d**) Cluster 3 group. Note. C1—Cluster 1; C2—Cluster 2; C3—Cluster 3; 5th minute of driving; 120th minute of driving; Statistically significant difference is indicated as * (*p* < 0.05) and ** (*p* < 0.001).

The measurement of applied interface pressure was significantly increased over the driving time in different seat pan areas (all *p* < 0.001). Further examination of interface pressure and differences between recording times, considering the subgroups individually, was reported in Figure 4. 

The greatest increases in applied average interface pressure occurred under the buttocks area (left buttock C1 = 35.6%; C2 = 46.2%; C3 = 42.1%; right buttock C1 = 46.9%; C2 = 80.7%; C3 = 60.7%; respectively). Increases in both thighs were reported after 120 min of steering the driving simulator as well (left thigh C1 = 40.6%; C2 = 37.9%; C3 = 48.3%; right thigh C1 = 48.4%; C2 = 74.1%; respectively). The lowest pressure comparing it with the previously mentioned lower limbs areas, yet with significant difference between two recording periods, were recorded at bolsters (left bolster C1 = 52.2%; C2 = 52.4%; C3 = 33.33% and right bolster C1 = 47.1%; C2 = 86.7%; C3 = 63.6%, respectively).

Although the differences in percentage were variated among the subgroups, Tukey’s HSD test did not indicate any substantial effect in the mean values by zones among groups, except for the left bolster (LB), where C1 generated a higher interface pressure C3 group (*p* = 0.021).

## 4. Discussion

### 4.1. Variation of Spatial and Temporal Data between Two Gait Cycles

The main goal of this study was to examine WS alterations between two walking periods and determine whether the changes of performed gait were made by discomfort developed over the driving time. The findings of this study indicate that participants’ self-selected WS has changed after the prolonged driving period. Interestingly, WS has increased for two groups (C1 and C2 groups). At the same time, 18% of the sample reduced WS (C3 group) after two hours of steering the driving simulator.

Higher WS directly contributes to higher muscle activity and dynamic musculoskeletal load and the greater kinetics values in the joints of the lower limbs [14,26]. Furthermore, a faster post-steering gait tends to affect the spatial and temporal variables [27], increasing the SL [28] and spending less time in the GCT [29]. Aiming attention to participants with faster WS after the long-time driving, the analysis of records indicated that the number of walking steps increased. At the same time, the TODST decreased.

Furthermore, the applied statistical methods showed that the new self-selected WS among the C2 group differs in shorter GCT, which is shorter, and in the TDST, increasing the SL (the mean value increased by 2.73 cm) after the prolonged driving. The prolonged driving process may trigger physiological and psychophysical responses, causing muscle exertion during the extended time driving and provoking participants to lose control of their self-selected comfort speed [14]. The different walking strategies might require more muscle activity of the lower limbs, more musculoskeletal dynamic load, and probably, dissimilar angular kinematics after two hours of the driving process [14,30,31]. 

Subgroup C3 opted for a different strategy, using a more cautious gait pattern, reducing the WS to counteract the postural instability [32]. The new walking pattern resulted in a prolonged GCT, TDST, and TODST. Decreasing the WS is not an obvious sign for the risk of falls since it is not clear whether individuals reduce it to prevent falling or decrease their movement as an adaptation to fear of falling [28]. Indeed, decreasing WS by 10 cm/s will increase the risk of falls by 7% [33]. The outcome indicated that the C3 reduced by 8.52 cm/s (on average) in the post-steering gait, potentially growing the risk of falling. A strong association was established between the risk of falls and decreased SL [28]. In their investigation, Verghese et al. [33] have concluded that reducing the SL by 3 cm will undoubtedly affect gait stability. It appears that the C3 participants, along with slower WS, tendentiously reduced the SL by on average 2.76 cm. Although the shorter SL was not statistically significant, it should be considered since it can disturb postural stability among the slower walking participants [33]. Additional factors responsible for the disturbance of walking strategies are fatigue in the lower limb muscles caused by long-time driving [34] and exposure to the higher amplitude of whole-body vibration, contributing to postural instability [35].

### 4.2. Influence of the Driving Venue and Long-Time Driving on Preferred Driving Postures 

Another element impacting drivers’ fatigability is the non-adjustable driving venue [10], limiting participants to select their comfortable vertical distances of the seat and steering wheel before the driving process. This issue might cause drivers to choose new, probably not their preferred, driving postures. The lack of a driving space can directly provoke discomfort among taller participants [36]. However, statistical analysis did not reveal any anthropometric difference among the participants. At the initial recording, self-selected driving strategies differ among slower (C3) and faster (C2) walkers in the knee and hip angles. The C3 participants choose to adopt more flexed angles. Noteworthy, the changes did not occur after 120 min of the driving process. Reposition of the preferred driving angles can signify felt discomfort in a specific part of the body. This indicates a strategy to reduce perceived discomfort in a body part, repositioning the body angle [37]. Subsequently, those changes in driving angles were not statistically significant. The only statistically essential difference was in the wrist among the C1 group, which could be recognised as perceived discomfort [3] caused by long-time driving.

### 4.3. Growth of Applied Interface Pressure Variables over the Driving Time

The first recordings of the covered area have indicated differences among the groups. It has been shown that participants with reduced WS (C3) covered a lesser seat pan area compared with the C2 (higher WS) group. This contrast in the covered area may be due to the different driving strategies adopted by these two groups. The prolonged driving process has subsequently influenced participants, and variation in the covered area was also established for the minute 120th (Figure 3b). Moreover, several authors suggested an interface pressure threshold value of 4.3 kPa below which capillary closure does not occur [21,22]. This value of interface pressure on the buttock skin is often used as a critical value of developing discomfort or in combination with long-term sitting deep tissue injuries such as pressure ulcers [38]. Interface pressure distribution over the lower limbs area did not exceed thresholds at the beginning of the driving process, except for the C1 group, where slightly higher values were reported under the thighs (LTH 3.12 ± 1.0 kPa; RTH 3.18 ± 1.3 kPa) area than recommended [23]. After two hours of steering the driving simulator, average interface pressure exceeded the defined threshold values (see Section 2.5) for the lower limbs area and reported higher values for each zone (Figure 4). At the end of the prolonged driving process, the most critical pressure values were under the thighs area, which could speed up perceived local body discomfort [23] and influence gait performance. As the result of the uniformity of the interface pressure distribution on the buttocks/thigh area pain/discomfort, or in the worst-case scenario, tissue ischemia might happen [10,39], which could speed up local body discomfort and influence performed walking strategies after the long-term driving. Furthermore, studying the interface pressure cannot predict the sub-dermal tissue strains and stresses [40] but could prevent skin injuries [12] and long-time deep tissue damages [40]. 

### 4.4. Limitations

This study investigates several strengths and limitations that need to be considered when interpreting its findings. Participants were composed of unprofessional, healthy, and young drivers. Such classification of non-professional drives may narrow a range of potential predictions, such as the influence of different anthropometric attributes and ages on preferred driving strategies, applied pressure, physical condition, among others. An additional limiting factor might be the influence of long-term exposure to a demanding virtual environment and the possible impact on their walking pattern. On the other side, the objective measurements indicated discomfort development and alteration in performed walking strategies. More significant consequences should be expected among older professional drivers based on the outcomes obtained in this study on healthy and young drivers. Studies investigating the discomfort developed during long-time driving and its influence on performed walking strategies were not identified. As the next step, it is important to investigate changes in angular kinematics and centre of the pressure variation to expand understanding of the influence of prolonged driving on gait pattern variation.

## 5. Conclusions

The present research showed that the applied interface pressure variables on the drivers’ seat pan have increased over the driving time. Uneven pressure distribution has been observed, with the highest prevalence under the buttocks and thighs area. Characterised groups have shown signs of discomfort, reaching the threshold value of average interface pressure over the total seat pan area at the 5th minute of driving. Average interface pressure under the buttocks and thighs area has exceeded threshold values after two hours of steering the driving simulator. Therefore, it can be expected that higher pressure values under the lower limbs might hamper blood circulation and cause local discomfort and therefore influence walking patterns.

There is strong evidence that physiological and psychophysical responses of the long-driving process and developed driving discomfort triggered alteration in WS, increasing it among most participants. Two hours of constant steering has influenced performed comfortable walking strategies among C3 participants. New walking strategy reflected in slower WS with prolonged GCT and Terminal and double support time. Additionally, preferred gait tended to reduce the SL new walking patterns among the C3 group.

## Figures and Tables

**Table 1:** Description of personal characteristics and driving experience (data is represented as mean ± standard deviation).

	C1	C2	C3	*p*-Value
Number of participants (M/F)	26 (15/11)	10 (3/7)	8 (4/4)	n.s.
WSD (cm/s)	2.8 ± 3.0	14.5 ± 3.3	−8.5 ± 6.3	<0.001
Age (years)	27.0 ± 4.8	27.0 ± 3.6	29.4 ± 5.6	n.s.
Body height (cm)	171.1 ± 8.1	168.1 ± 8.6	171.1 ± 9.2	n.s.
Weight (kg)	70.9 ± 15.4	66.1 ± 14.0	62.9 ± 11.0	n.s.
BMI (kg/m^2^)	24.2 ± 4.3	23.3 ± 3.8	21.3 ± 2.5	n.s.
Driving experience (years)	7.2 ± 4.6	8.6 ± 6.1	7.1 ± 2.8	n.s.
Driving per week (hours)	5.1 ± 4.2	4.7 ± 4.2	2.5 ± 2.9	n.s.

Note. M—Male; F—Female; WSD—walking speed differences (differences = post-walking speed—pre-waling speed); BMI—Body mass index; n.s.—Not statistically significant.

**Table 2:** Spatial and temporal data for pre- and post-steering gait (data reported in the mean ± standard deviation).

Spatiotemporal Variable	Period	C1	C2	C3
WS (cm/s)	Pre	91.03 ± 9.79	96.61 ± 16.91	97.80 ± 14.62
	Post	93.88 ± 10.84	111.08 ± 15.96	89.28 ± 17.48
*p*-value		<0.001	<0.001	<0.001
SL (cm)	Pre	54.57 ± 5.09	54.93 ± 5.60	57.56 ± 5.12
	Post	55.76 ± 8.36	57.66 ± 7.73	54.80 ± 4.72
*p*-value		n.s.	n.s.	n.s.
CA (steps/min)	Pre	101.05 ± 7.73	104.91 ± 9.50	102.40 ± 7.65
	Post	104.55 ± 9.48	113.16 ± 7.14	98.30 ± 8.64
*p*-value		0.002	<0.001	0.041
SST (s)	Pre	0.44 ± 0.04	0.43 ± 0.04	0.44 ± 0.05
	Post	0.43 ± 0.04	0.41 ± 0.03	0.45 ± 0.04
*p*-value		n.s.	n.s.	n.s.
IDST (s)	Pre	0.17 ± 0.04	0.15 ± 0.04	0.15 ± 0.04
	Post	0.16 ± 0.03	0.13 ± 0.03	0.17 ± 0.06
*p*-value		n.s.	n.s.	n.s.
TDST (s)	Pre	0.17 ± 0.03	0.18 ± 0.04	0.16 ± 0.02
	Post	0.16 ± 0.03	0.14 ± 0.03	0.20 ± 0.08
*p*-value		n.s.	<0.001	0.002
TODST (s)	Pre	0.34 ± 0.06	0.33 ± 0.05	0.31 ± 0.06
	Post	0.32 ± 0.05	0.28 ± 0.05	0.37 ± 0.10
*p*-value		0.002	<0.001	<0.001
GCT	Pre	1.20 ± 0.08	1.21 ± 0.12	1.15 ± 0.13
	Post	1.17 ± 0.10	1.10 ± 0.09	1.23 ± 0.10
*p*-value		n.s.	<0.001	0.010
STF (%)	Pre	64.37 ± 2.30	62.45 ± 4.77	65.36 ± 1.87
	Post	64.00 ± 2.22	62.12 ± 3.50	65.65 ± 4.05
*p*-value		n.s.	n.s.	n.s.
SWF (%)	Pre	35.63 ± 2.30	37.55 ± 4.77	34.64 ± 1.87
	Post	36.00 ± 2.22	37.88 ± 3.50	34.35 ± 4.05
*p*-value		n.s.	n.s.	n.s.

Note. C1—Cluster 1; C2—Cluster 2; C3—Cluster 3; WS—Walking speed; SL—Step length; CA—Cadence; SST—Single support time; IDST—Initial double support time; TDST—Terminal double support time; TODST—Total double support time; GCT—Gait cycle time; STF—Stance phase; SWF—Swing phase; n.s.—Not statistically significant. Statistically significant difference is between pre-and post-steering gait indicated as (*p* < 0.05) or (*p* < 0.001).

## Data Availability

Data is contained within the article.
